# Expanding CRISPR/Cas9 Genome Editing Capacity in Zebrafish Using SaCas9

**DOI:** 10.1534/g3.116.031914

**Published:** 2016-06-16

**Authors:** Yan Feng, Cheng Chen, Yuxiang Han, Zelin Chen, Xiaochan Lu, Fang Liang, Song Li, Wei Qin, Shuo Lin

**Affiliations:** * Laboratory of Chemical Genomics, School of Chemical Biology and Biotechnology, Peking University Shenzhen Graduate School, 518055, China; † Department of Molecular, Cell and Developmental Biology, University of California, Los Angeles, California 90095

**Keywords:** CRISPR/Cas9, SaCas9, KKH SaCas9 variant, gene editing, zebrafish

## Abstract

The type II CRISPR/Cas9 system has been used widely for genome editing in zebrafish. However, the requirement for the 5′-NGG-3′ protospacer-adjacent motif (PAM) of Cas9 from *Streptococcus pyogenes* (SpCas9) limits its targeting sequences. Here, we report that a Cas9 ortholog from *Staphylococcus aureus* (SaCas9), and its KKH variant, successfully induced targeted mutagenesis with high frequency in zebrafish. Confirming previous findings, the SpCas9 variant, VQR, can also induce targeted mutations in zebrafish. Bioinformatics analysis of these new Cas targets suggests that the number of available target sites in the zebrafish genome can be greatly expanded. Collectively, the expanded target repertoire of Cas9 in zebrafish should further facilitate the utility of this organism for genetic studies of vertebrate biology.

Targeted genome engineering offers new genetic tools for understanding biological processes, and for developing therapeutics of human diseases. Following the development of Zinc Finger (ZFs) and transcription activator-like effector (TALEs) technology (Doyon *et al.* 2008; Huang *et al.* 2011; Meng *et al.* 2008), the clustered regularly interspaced short palindromic repeat/CRISPR-associated (CRISPR/Cas) system has emerged as the current gene editing tool of choice. CRISPR/Cas system has the advantages of ease of handling, low cost, and universal applicability in different cell types and organisms. CRISPR/Cas can be classified into six types based on the presence of ‘‘signature genes’’ (Makarova *et al.* 2011, 2015; Shmakov *et al.* 2015; Wright *et al.* 2016). Among them, Cas9 from *Streptococcus pyogenes* (SpCas9), which belongs to the type II CRISPR/Cas system, has been demonstrated to be effective in inducing targeted DNA double strand breaks (DSBs) in a variety of organisms (Chang *et al.* 2013; Cong *et al.* 2013; Dickinson *et al.* 2013; Friedland *et al.* 2013; Gratz *et al.* 2013; Hwang *et al.* 2013; Jinek *et al.* 2013; Mali *et al.* 2013; Qin *et al.* 2015; Shalem *et al.* 2014; Wang *et al.* 2014; Yang *et al.* 2013a). SpCas9 nuclease DNA sequence specificity relies on a guide RNA with a protospacer-adjacent motif (PAM) sequence at the 3′ end of a 20-bp target sequence. The most widely used SpCas9 recognizes a short 5′-NGG-3′ PAM. Since PAM sequences are different in different CRISPR/Cas systems, alternative PAMs would provide more flexibility for targeting strategies such as precise knock-in mutations.

Recently, Cas9 orthologs with distinct DNA binding specificity and PAM recognition, including *Neisseria meningitidis* (NmCas9), *Streptococcus thermophilus*1 (St1Cas9), and *Staphylococcus aureus* (SaCas9) have been applied for genome editing in human cells (Hou *et al.* 2013; Karvelis *et al.* 2013; Ran *et al.* 2015). Among them, Cas9 from *S. aureus* (SaCas9) is smaller, and has a longer PAM of 5′-NNGRRT-3′sequence. These features allow easier deliver by viral expression vectors, and higher sequence specificity, which would be more desirable for therapeutic applications. Recently, a SaCas9 variant (KKH SaCas9) with partially relaxed 5′-NNNRRT-3′ PAM specificities has been demonstrated to show robust genome editing activities in human cells, which further increases the SaCas9 targeting range (Kleinstiver *et al.* 2015a).

Here, we demonstrate that SaCas9, with its KKH SaCas9 variant, can edit the zebrafish genome with high targeting efficiency. This increases the frequency of available target sites, and expands the utility of CRISPR/Cas9 in zebrafish by targeting those previously inaccessible Cas9 sites in the genome.

## Materials and Methods

### Zebrafish husbandry and breeding

Wild type Tu fish and *tg(mylpfa:EGFP)* transgenic fish strains were raised and maintained at 28.5° in a circulating system. Zebrafish embryos were acquired from in-tank breeding. Development of embryos was staged by standard morphological criteria (Kimmel *et al.* 1995). All zebrafish experiments were approved by the Institutional Animal Care and Use Committee (IACUC) of Peking University. The reference from IACUC of Peking University is LSC-ZhangB-1.

### Plasmid construction and RNA synthesis

The full-length humanized NLS-SaCas9-NLS product was cloned from plasmid (Addgene#61591), and subcloned into the pCS2+ vector. pX601-AAV-CMV::NLS-SaCas9-NLS-3xHA-bGHpA;U6::*Bsa*I-sgRNA was a gift from Feng Zhang. SaCas9 and SpCas9 variant VQR were mutated on the basis of NLS-SaCas9-NLS and pT7-nls-zCas9-nls plasmids (Liu *et al.* 2014), respectively, using Vazyme Mut Express II Fast Mutagenesis Kit V2. After linearization by either *Not*I (pCS2-nls-hSaCas9-nls) or *Xba*I (pT7-nls-zCas9-nls), capped mRNA was synthesized using a mMESSAGE mMACHINE Sp6 or T7 kit, and purified using an RNeasy FFPE kit (Qiagen). All gRNAs templates in this study were prepared according to the cloning-independent gRNA generation method, and all sites are listed in Supplemental Material, Table S1 (Bassett *et al.* 2013). gRNAs were transcribed *in vitro* using the T7 MAXIscript Kit (Ambion), and purified using an RNeasy FFPE kit (Qiagen). Table S2 lists all the oligos used in this study.

### Zebrafish microinjection, T7EI assays, and Sanger sequencing

A solution (1–2 nl) containing Cas9 mRNA (300 ng/μl) and gRNA (30 ng/μl) was coinjected into one-cell-stage zebrafish embryos. Injected embryos were incubated at 28.5° for examination of phenotypes. After 2 d post fertilization (dpf), embryos that developed normally were collected for genotyping. Genomic DNA was extracted from pools of six randomly collected embryos by alkaline lysis buffer-based DNA extraction. Targeted genomic loci were amplified from genomic DNA, and then cloned into the pEASY-T1 vector (Transgene) for sequencing. *T7E*1 assays were performed as previously described for zebrafish (Hwang *et al.* 2013). The digested samples were analyzed through a 2% agarose gel. Quantification was based on relative band intensity using Quantity One software (Bio-Rad). All experiments were repeated three times.

### Imaging

Zebrafish embryos were anesthetized with 0.03% Tricaine (Sigma-Aldrich), and mounted in 4% methylcellulose. Photographs were taken by a Zeiss Axio Imager Z1 microscope, and processed by Adobe Photoshop CC software.

### Annotation of CRISPR target sites in coding exons

We searched for all potential CRISPR target sites of NGG, NGA, NNGRRN, NNGRRT, NNNRRN, and NNNRRT on both strands of the zebrafish genome (danRer10), and marked their chromosomal positions. Then, we produced a BED file to show all these PAM sites in exons as annotated by the UCSC browser.

### Data availability

The authors state that all data necessary for confirming the conclusions presented in the article are represented fully within the article.

## Results and Discussion

### Gene editing in zebrafish using SaCas9

**Figure 1:**
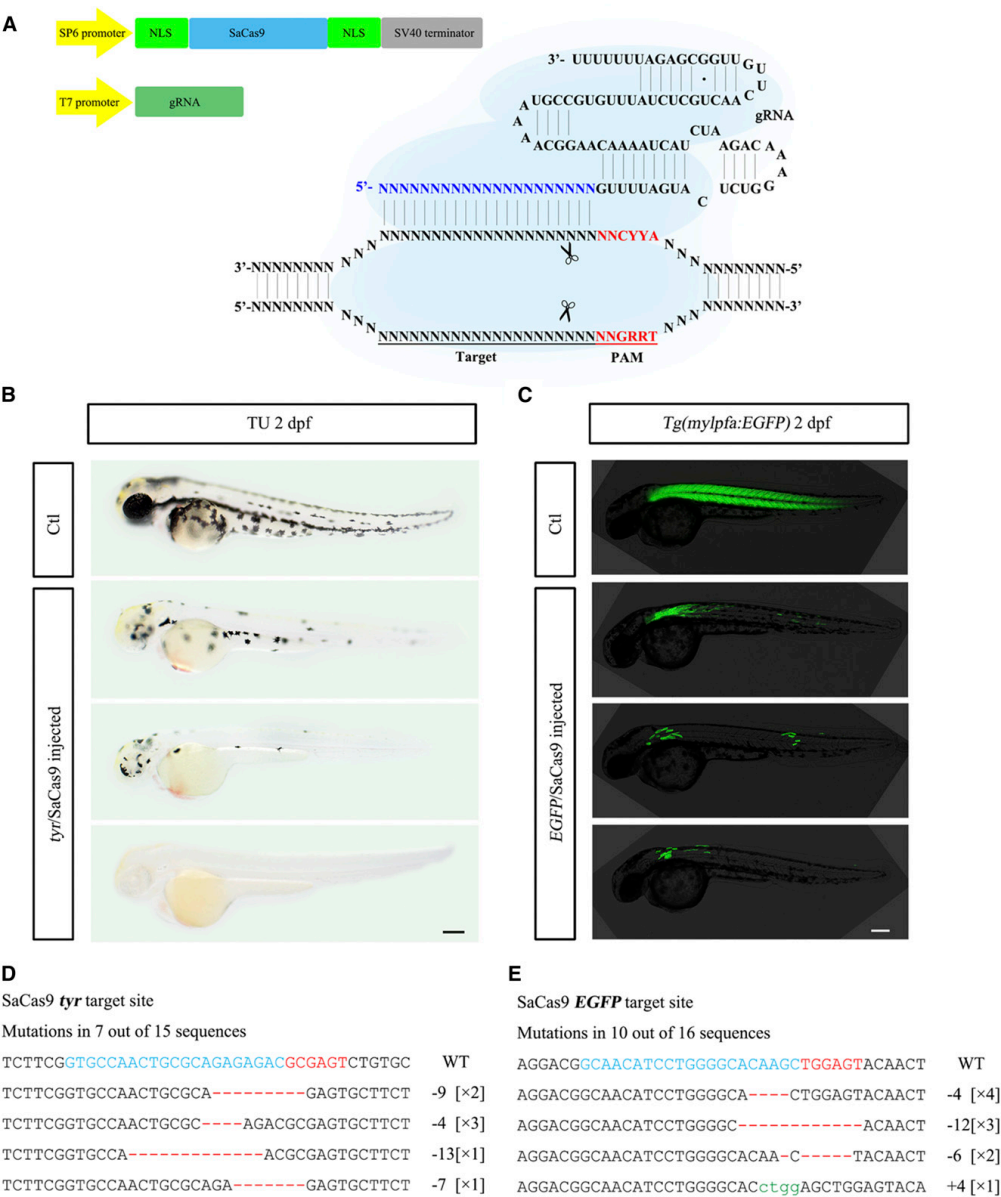
Gene disruption by SaCas9. (A) Schematic representation of the CRISPR/SaCas9 system, including SaCas9 and the gRNA. SaCas9 flanked by two NLS signals is driven by SP6 promoter, and the gRNA is driven by T7 promoter. The PAM is highlighted in red. The target site is labeled by blue color. (B) Phenotype of embryos by targeting the *tyr* gene. Scale bar = 500 μm. (C) Phenotype of embryos by targeting the *EGFP* transgene. Scale bar = 500 μm. (D-E**)** Sequencing results at the *tyr* and *EGFP* targets. Target sequence (blue), PAM region (red), deletion (red dashes) and insertions (lower case letters in green) are indicated. The numbers of mutant alleles are indicated in [×] brackets.

First, we engineered SaCas9 to contain a NLS sequence in the pCS2+ expression vector, and used a simple one-step cloning-free PCR method to generate gRNA template for RNA transcription *in vitro* (Figure 1A) (Bassett *et al.* 2013). Although SaCas9 can target all 5′-NNGRR-3′ PAMs, it cleaves the sequence of 5′-NNGRRT-3′ most efficiently (Ran *et al* 2015). We therefore selected this type of PAM sequence from the zebrafish genome for initial testing. Since pigmentation defect is a convenient phenotype indication for mutation in the tyrosinase (*tyr*) gene, which encodes an enzyme that converts tyrosine into melanin (Camp and Lardelli 2001), we designed a gRNA targeting *tyr*. After coinjection of *tyr* gRNA and SaCas9 mRNA into one-cell-stage zebrafish embryos, pigmentation reduction was observed in 42 embryos (*n* = 73), some of which totally lacked pigmentation (Figure 1B). Injected embryos developed normally, no obvious toxicity was observed. We randomly selected six embryos, extracted genomic DNA from pools of embryos and assessed the mutagenesis rates using the T7 endonuclease I (*T7E*I) assay and further confirmed indels at the expected sites by sequencing (Figure 1D).

To test if SaCas9/gRNA can be used generally in zebrafish, we constructed eight additional gRNAs, one targeting transgene EGFP and others targeting endogenous genes. When EGFP SaCas9/gRNA was injected into *tg* (*mylpfa*:*EGFP*) zebrafish eggs, loss of EGFP expression was observed in muscle cells (Figure 1C). For other specific sites, indels were detected in all injected embryos with a frequency of 13–76%. Again, the indels were sequencing confirmed at the expected genomic loci (Figure 1E and Figure S1). Taken together, these results demonstrate that SaCas9 can effectively edit genome in zebrafish.

### Gene editing in zebrafish using KKH SaCas9

**Figure 2:**
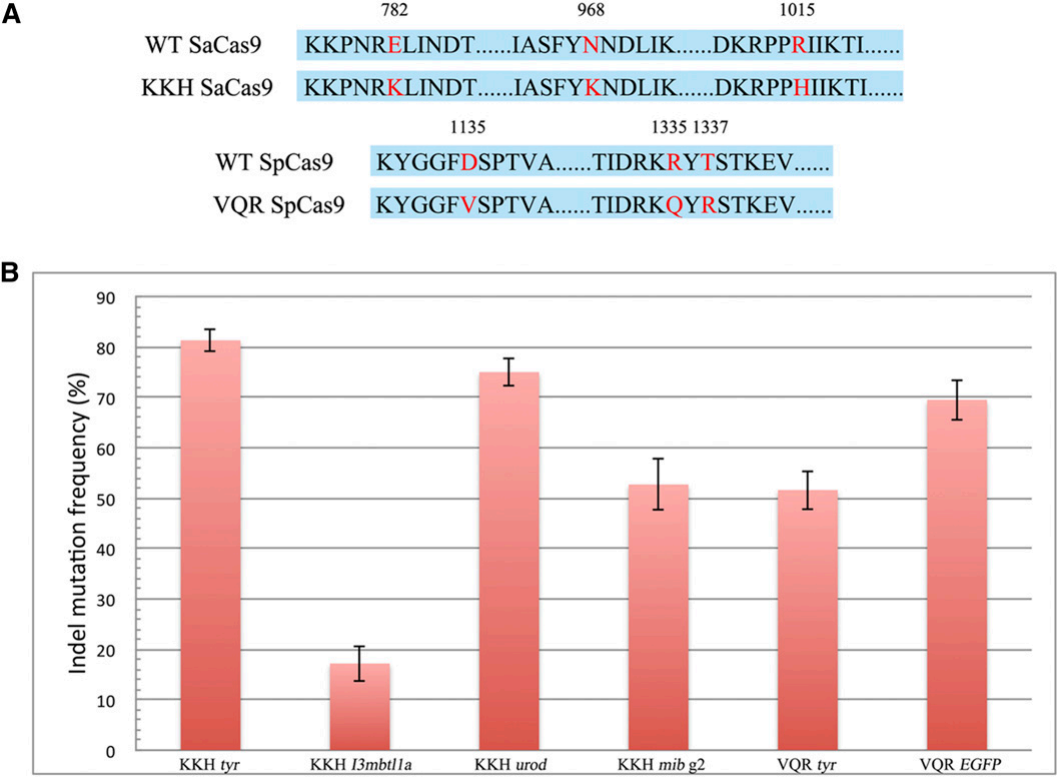
KKH SaCas9- and VQR SpCas9-mediated genome editing in zebrafish. (A) PAM-interacting domain for KKH SaCas9 and VQR SpCas9 in amino acid sequence comparison with SaCas9 and SpCas9, respectively. Variable amino acid residues are highlighted in red. (B) Activity of KKH SaCas9 and VQR SpCas9 targeting zebrafish endogenous genes. Quantification is based on *T7E*1 assay. Error bars represent SEM, *n* = 6 embryos. Sequencing data of these targeted indels is included in Figure S2.

Native SaCas9 recognizes a longer 5′-NNGRRT-3′ PAM, which occurs on average every 32 bps of random DNA. Obtaining variants with relaxed or partially relaxed specificity to expand the repertoire of gene editing tools in zebrafish is desirable. Toward this goal, we investigated two recently described SaCas9 and SpCas9 variants with different specificities (Figure 2A) (Kleinstiver *et al.* 2015a, 2015b). One SaCas9 variant is called KKH SaCas9, which can target 5′-NNNRRT-3′ PAM in human genome (Kleinstiver *et al.* 2015a). We evaluated the activity of KKH SaCas9 by targeting four endogenous genes in zebrafish, and observed indels in all of them (Figure 2B and Figure S2). Similarly, we evaluated activity of the SpCas9 VQR variant with an NGA PAM, which has been shown previously to induce indels in zebrafish and *Caenorhabditis elegans* (Bell *et al.* 2016; Kleinstiver *et al.* 2015b), and obtained expected indels at the two predicted sites (Figure 2B and Figure S2). Collectively, these results demonstrate that both KKH SaCas9 and VQR SpCas9 variants are functional in zebrafish. Since 5′-NNNRRT-3′ represents a relaxed PAM, it is conceivable that the off-target rate might be increased. In zebrafish, this potential problem could be addressed by analyzing the linkage between the targeted mutation, and the specific phenotype in large numbers of individual fish through breeding multiple generations.

The SpCas9 system is an efficient tool for genome editing in many organisms including bacteria, yeast, human cells, *C. elegans*, zebrafish, and mice (Hsu *et al.* 2014; Sternberg and Doudna 2015). Due to the restriction of having to use the 5′-NGG-3′ PAM required by SpCas9, it is challenging to find target sites with high efficiency in a specific genome locus. Improving the capacity to target very specific loci is highly desirable for DNA homology-directed gene repair, which is most efficient when a DSB is placed within 10–20 bp of a desired alteration (Elliott *et al.* 1998; Findlay *et al* 2014; Yang *et al.* 2013b). Here, we demonstrate that using SaCas9 and its variant enable genome editing in a significantly wider range in zebrafish. Since Cas9-based gene editing is most commonly applied to induce or repair mutations in coding exons of a gene, we produced a BED file to annotate six types of target sites (NGG, NGA, NNGRRN, NNGRRT, NNNRRN, and NNNRRT) on the UCSC gene browser.

One of the key issues in using CRISPR/Cas9 system successfully is to assure high activity. First, similar to the zebrafish-codon-optimized SpCas9, which increases gRNA targeting efficiency from ∼4% to ∼39% (Liu *et al.* 2014), we recommend constructing a zebrafish codon-optimized SaCas9 for future study. Second, it is also possible that direct SaCas9 protein injections could further increase efficiency (Burger *et al.* 2016; Gagnon *et al.* 2014). Finally, as modifying the SpCas9/gRNA structure by extending the duplex length has been shown to improve knockout efficiency (Dang *et al.* 2015), we speculate that the same strategy could be applied to SaCas9/gRNA structure in zebrafish.

## Supplementary Material

HTML Page - index.htslp
