# Supplementary material for: Expanding CRISPR/Cas9 Genome Editing Capacity in Zebrafish Using SaCas9
Source: G3 (Bethesda). 2016 Jun 16;6(8):2517–21. doi: 10.1534/g3.116.031914 (PMC4978904; doi:10.1534/g3.116.031914)
Supplement: HTML Page - index.htslp [file supp_6_8_2517__index.html]

Expanding CRISPR/Cas9 Genome Editing Capacity in Zebrafish Using SaCas9 — HTML Page - index.htslp 

# Expanding CRISPR/Cas9 Genome Editing Capacity in Zebrafish Using SaCas9

## Supplemental Material for Feng, *et al*, 2016

**Files in this Data Supplement:**

- Figure S1 - Targeting efficiency measured by *T7E*1 assays and sequencing at SaCas9 target sites. (.doc, 1.18 MB)
- Figure S2 - Sequencing the target sites or phenotype analysis of KKH SaCas9/gRNA and VQR SpCas9/gRNA injected embryos. (.docx, 358 KB)
- Table S1 - Target sites in this study. (.docx, 13 KB)
- Table S2 - Primers used in this study. (.docx, 14 KB)
