# Supplementary material for: Expanding CRISPR/Cas9 Genome Editing Capacity in Zebrafish Using SaCas9
Source: G3 (Bethesda). 2016 Jun 16;6(8):2517–21. doi: 10.1534/g3.116.031914 (PMC4978904; doi:10.1534/g3.116.031914)
Supplement: HTML Page - index.htslp [file supp_g3.116.031914_FigureS1.doc]

**
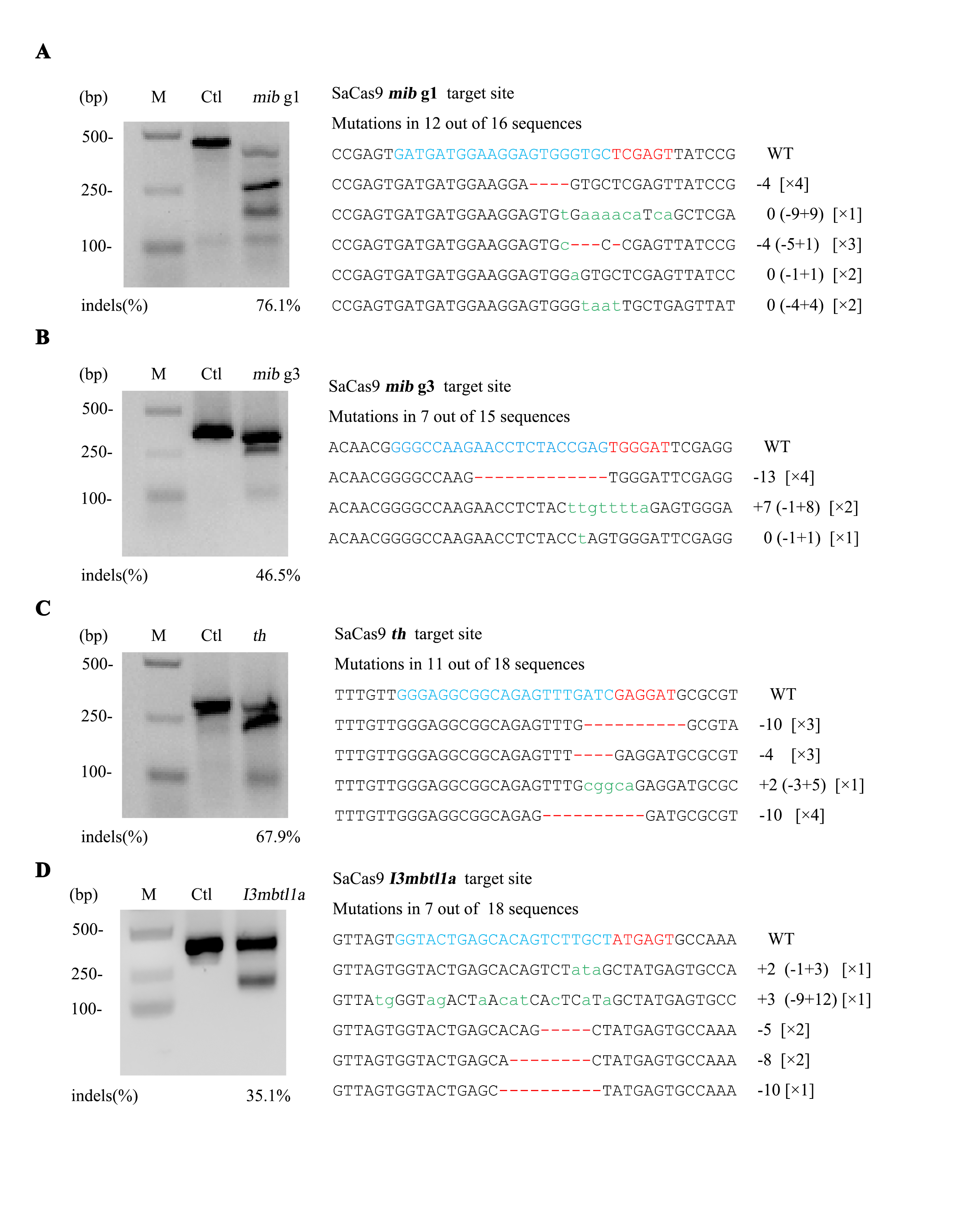
**

**
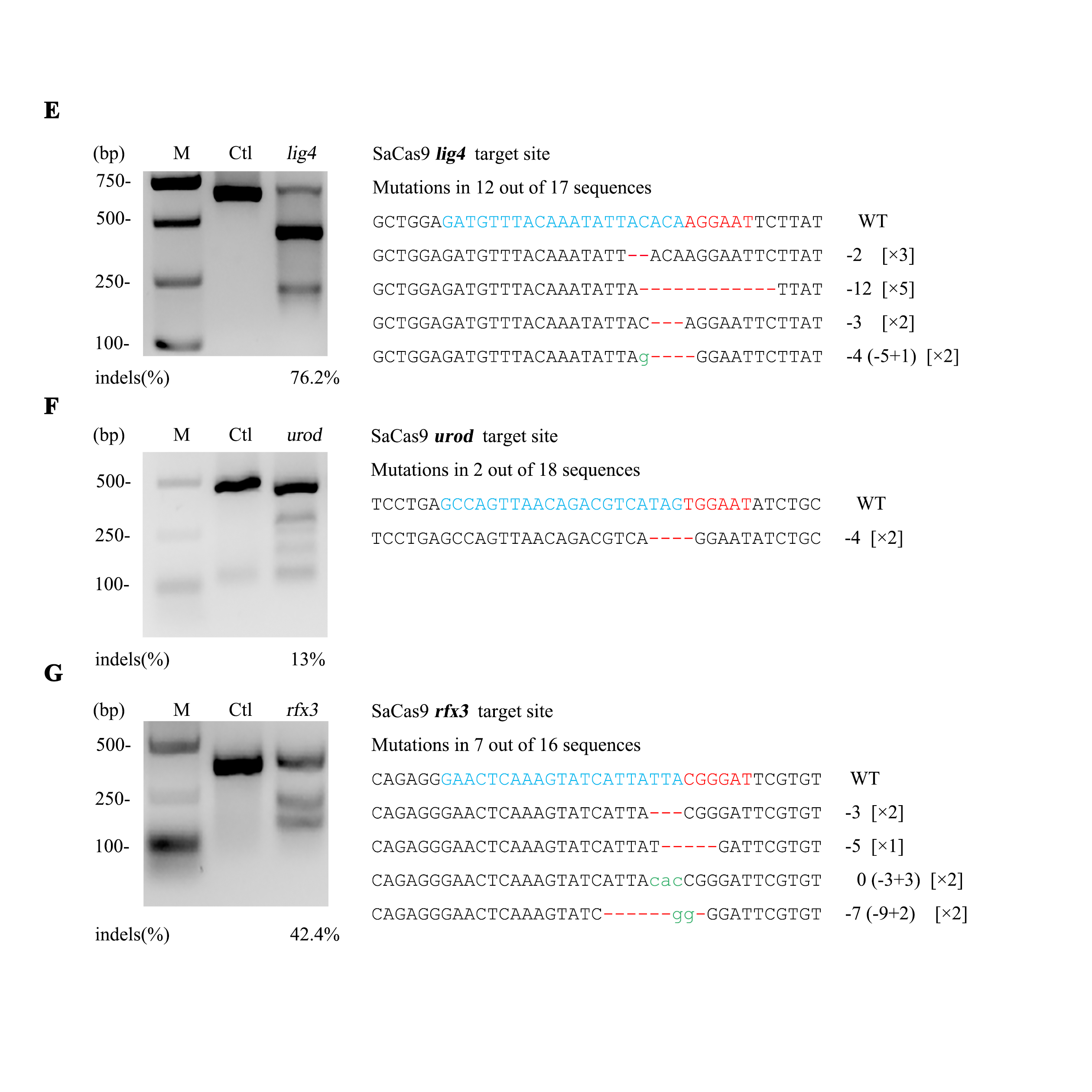
**

**Figure S1** Targeting efficiency measured by *T7E*1 assays and sequencing at SaCas9 target sites.

A-G: *T7E*1 and sequencing results of SaCas9 *mib* g1, *mib* g3, *th*, *I3mbtl1a*, *lig4*, *urod*, *rfx3* sites.
