# Supplementary material for: Expanding CRISPR/Cas9 Genome Editing Capacity in Zebrafish Using SaCas9
Source: G3 (Bethesda). 2016 Jun 16;6(8):2517–21. doi: 10.1534/g3.116.031914 (PMC4978904; doi:10.1534/g3.116.031914)
Supplement: HTML Page - index.htslp [file supp_g3.116.031914_FigureS2.docx]

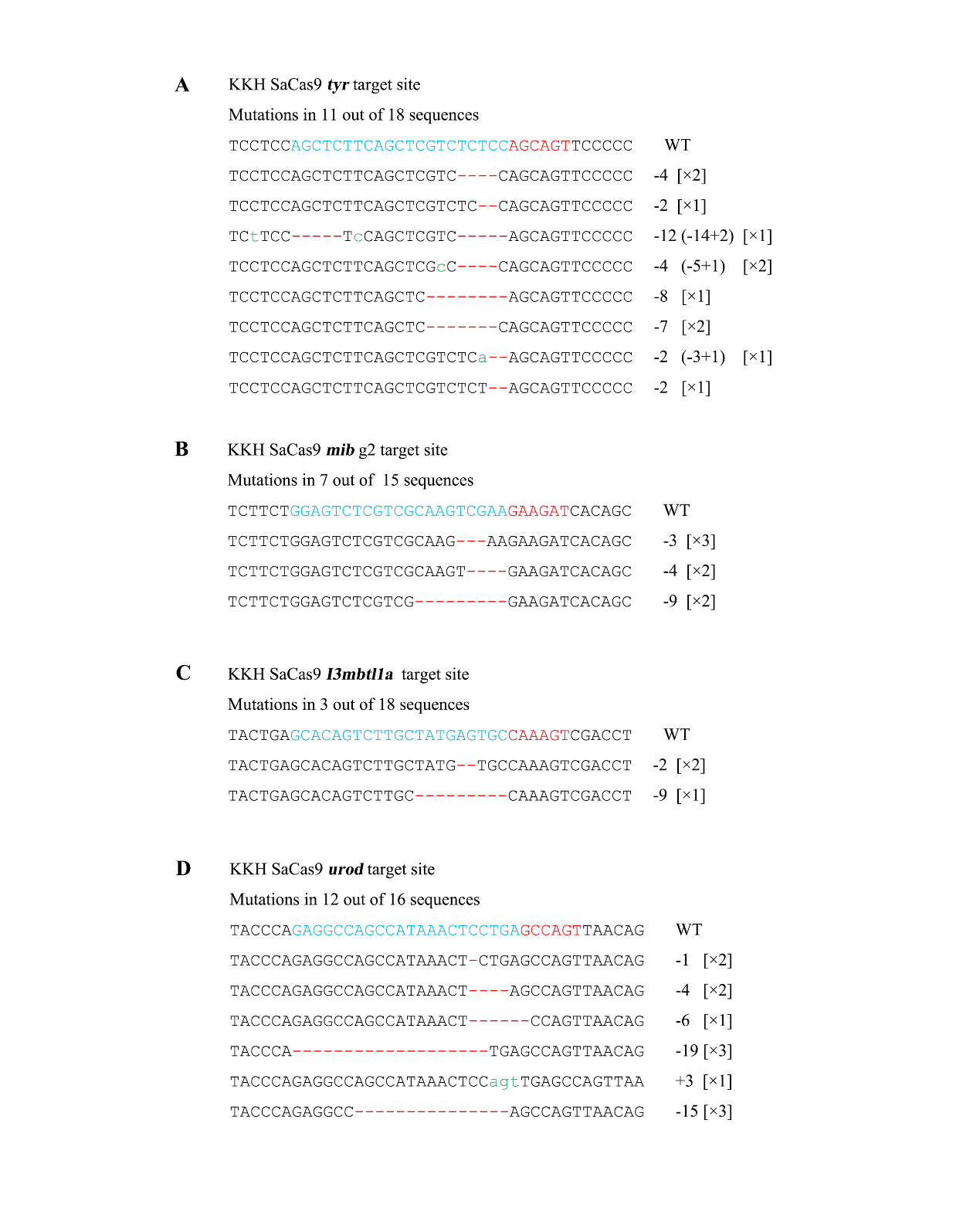
**
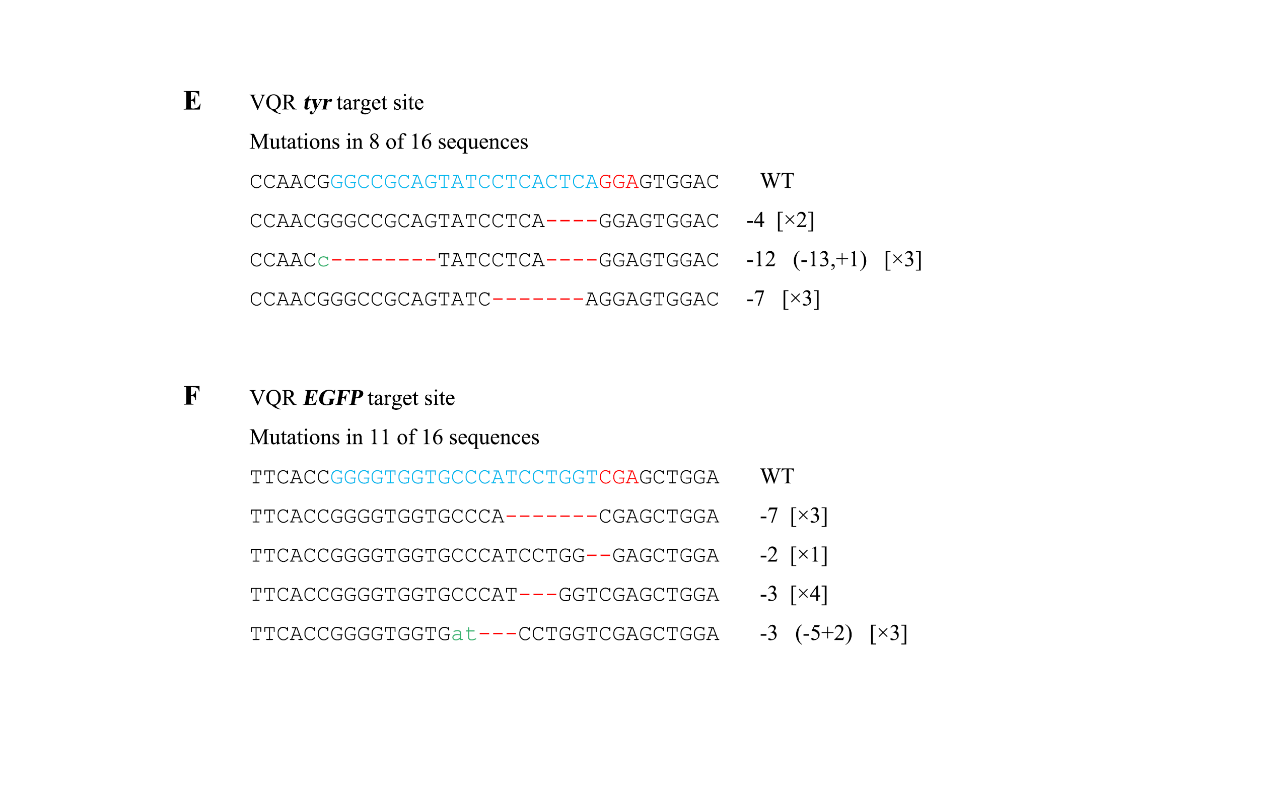
**

**Figure S2** Sequencing the target sites or phenotype analysis of KKH SaCas9/gRNA and VQR SpCas9/gRNA injected embryos. Target sequence (blue), PAM region (red), deletion (red dashes) and insertions (lower case letters in green) are indicated, respectively. The numbers of mutant alleles are indicated in [x] brackets.
