# Supplementary material for: Expanding CRISPR/Cas9 Genome Editing Capacity in Zebrafish Using SaCas9
Source: G3 (Bethesda). 2016 Jun 16;6(8):2517–21. doi: 10.1534/g3.116.031914 (PMC4978904; doi:10.1534/g3.116.031914)
Supplement: HTML Page - index.htslp [file supp_g3.116.031914_TableS1.docx]

**Table S1 Target sites in this study.**

| Gene name | Target sites |
| --- | --- |
| *tyr* for SaCas9 | GTGCCAACTGCGCAGAGAGACGCGAGT |
| *tyr* for KKH SaCas9 | AGCTCTTCAGCTCGTCTCTCCAGCAGT |
| *tyr* for VQR SpCas9 | GGCCGCAGTATCCTCACTCAGGA |
| *th* for SaCas9 | GGGAGGCGGCAGAGTTTGATCGAGGAT |
| *urod* for KKH SaCas9 | GAGGCCAGCCATAAACTCCTGAGCCAGT |
| *urod* for SaCas9 | GCCAGTTAACAGACGTCATAGTGGAAT |
| *lig4* for SaCas9 | GATGTTTACAAATATTACACAAGGAAT |
| *rfx3* for SaCas9 | GAACTCAAAGTATCATTATTACGGGAT |
| *I3mbtl1* for SaCas9 | GGTACTGAGCACAGTCTTGCTATGAGT |
| *I3mbtl1* for KKH SaCas9 | GCACAGTCTTGCTATGAGTGCCAAAGT |
| *mib* g1 for SaCas9 | GATGATGGAAGGAGTGGGTGCTCGAGT |
| *mib* g2 for KKH SaCas9 | GGAGTCTCGTCGCAAGTCGAAGAAGAT |
| *mib* g3 for SaCas9 | GGGCCAAGAACCTCTACCGAGTGGGAT |
| *EGFP* for SaCas9 | GCAACATCCTGGGGCACAAGCTGGAGT |
| *EGFP* for VQR SpCas9 | GGGGTGGTGCCCATCCTGGTCGA |

Red colors mean PAM sequences.
