# Supplementary material for: Expanding CRISPR/Cas9 Genome Editing Capacity in Zebrafish Using SaCas9
Source: G3 (Bethesda). 2016 Jun 16;6(8):2517–21. doi: 10.1534/g3.116.031914 (PMC4978904; doi:10.1534/g3.116.031914)
Supplement: HTML Page - index.htslp [file supp_g3.116.031914_TableS2.docx]

**Table S2 Primers used in this study.**

| Name | Sequence of the primer (5’-3’) |
| --- | --- |
| *tyr* detect | F: CTGGTGTCCGACCTTCCCAACGGG |
|  | R: TTCCGGATCTCATGCTCCCAAAACA |
| *th* detect | F: CTTTCGACAGTCTCAGAAGTTTG |
|  | R: CTGTGTGTCAAAGACCAAATACC |
| *urod* detect | F: AATAGATGCAACTTTGCTGA |
|  | R: GTTCTAAATTGTGCTGCAAAT |
| *lig4* detect | F: TCCAGATGCACCAGAATTCT |
|  | R: CATGAGGGCTAAAGACATTG |
| *rfx3* detect | F: TTGGACGTGGCAGAAGATCG |
|  | R: CAGCAGCTCAAACACTCTAA |
| *I3mbtl1* detect | F: AAAACCAGCCAACCAGCCTA |
|  | R: TGGTGTCCTGGGGTTTCAG |
| *mib* target 1 detect | F1: CCATATGTAAGACGAACCAG |
|  | R1: AAGGTGAGAGATGAAGTCAA |
| *mib* target 2 detect | F2: TCCTTACTGACGGCTGTTGC |
|  | R2: GATGAGCACAGGAAGGTGAG |
| *mib* target 3 detect | F3: GTCCATAGTGCATCATTTGG |
|  | R3: AAGGCCTGTTAACTGCAGTC |
| *EGFP* detect | F: CCATCTTCTTCAAGGACGAC |
|  | R: TGCTCAGGTAGTGGTTGTCG |
| VQR SpCas9  R1335Q,T1337R double mutation | F:ATAGAAAGcaGTACAgaTCCACTAAGGAGGTGCTGGACGCTACACTGA |
|  | R:CCTTAGTGGAtcTGTACtgCTTTCTATCGATTGTAGTGTCGAAGTACTTG |
| VQR SpCas9  D1135V | F:GGAGGCTTTGtCTCTCCCACTGTGGCATACTCCGTCCTGGTG |
|  | R:AGTGGGAGAGaCAAAGCCTCCATACTTCTTAGGGTCCCAGTC |
| BamHI-SaCas9 sense | ggatccATGGCCCCAAAGAAGAAGCGGAAGGTC |
| XhoI-SaCas9 antisense | ctcgagttaCTTTTTCTTTTTTGCCTGGCCGGCC |
| KKH SaCas9 E782K | F:GCCTAATAGAAAGCTGATTAACGACACCCTGTACTCCACCCG  R:TTAATCAGCTTTCTATTAGGCTTCTTGTCCACCCGGTGGCTG |
| KKH SaCas9 N968K | F:CCTTCTACAAAAACGATCTGATCAAGATCAACGGCGAGCTGT  R:TCAGATCGTTTTTGTAGAAGGAGGCGATAAACTCGGCCTGGT |
| KKH SaCas9 R1015H | F:AGGCCCCCCCACATCATTAAGACAATCGCCTCCAAGACCCAG  R:CTTAATGATGTGGGGGGGCCTCTTGTCGTTCATGTTTTCCAG |
